# Quantification of the impact of PSI:Biology according to the annotations of the determined structures

**DOI:** 10.1186/1472-6807-13-24

**Published:** 2013-10-21

**Authors:** Paul J DePietro, Elchin S Julfayev, William A McLaughlin

**Affiliations:** 1 Department of Basic Science, The Commonwealth Medical College, 525 Pine Street, Scranton, PA 18509, USA

**Keywords:** Protein Structure Initiative, Structural genomics, Protein annotations, Protein annotation, Scientific partnerships, Structure to function relationships

## Abstract

**Background:**

Protein Structure Initiative:Biology (PSI:Biology) is the third phase of PSI where protein structures are determined in high-throughput to characterize their biological functions. The transition to the third phase entailed the formation of PSI:Biology Partnerships which are composed of structural genomics centers and biomedical science laboratories. We present a method to examine the impact of protein structures determined under the auspices of PSI:Biology by measuring their rates of annotations. The mean numbers of annotations per structure and per residue are examined. These are designed to provide measures of the amount of structure to function connections that can be leveraged from each structure.

**Results:**

One result is that PSI:Biology structures are found to have a higher rate of annotations than structures determined during the first two phases of PSI. A second result is that the subset of PSI:Biology structures determined through PSI:Biology Partnerships have a higher rate of annotations than those determined exclusive of those partnerships. Both results hold when the annotation rates are examined either at the level of the entire protein or for annotations that are known to fall at specific residues within the portion of the protein that has a determined structure.

**Conclusions:**

We conclude that PSI:Biology determines structures that are estimated to have a higher degree of biomedical interest than those determined during the first two phases of PSI based on a broad array of biomedical annotations. For the PSI:Biology Partnerships, we see that there is an associated added value that represents part of the progress toward the goals of PSI:Biology. We interpret the added value to mean that team-based structural biology projects that utilize the expertise and technologies of structural genomics centers together with biological laboratories in the community are conducted in a synergistic manner. We show that the annotation rates can be used in conjunction with established metrics, i.e. the numbers of structures and impact of publication records, to monitor the progress of PSI:Biology towards its goals of examining structure to function connections of high biomedical relevance. The metric provides an objective means to quantify the overall impact of PSI:Biology as it uses biomedical annotations from external sources.

## Background

Protein Structure Initiative: Biology (PSI:Biology) determines the structures of proteins on a large scale to reveal their biological functions. Scientific partnerships (PSI:Biology Partnerships) are formed between scientists in the structural genomics centers and those in biological laboratories of the community. Through the work of the partnerships, broad and challenging biological questions are addressed [1]. Example areas of study include metagenomics and microbiomes, whole genomes of organisms and organelles, and proteins along pathways as described by systems biology approaches. The technologies and expertise available in the structural genomics centers enable protein structures to be determined in high-throughput [2,3].

Systematic approaches are utilized for structure determination through the PSI, and the methodological pipeline starts with the selection of protein targets [4,5]. Summaries of the target selection processes are described in the Structural Biology Knowledgebase [6]. One approach to target selection is to choose representative sequences from protein families. An example application of that approach is to obtain the structures of unique folds. That application has been demonstrated through the attainment of representative structures of proteins with unknown folds, as was done in the first two phases of PSI (PSI:1&2). The project is subsequently continued into the third phase of PSI, PSI:Biology.

The resultant structures of unique protein folds are used directly in further studies or they are leveraged to obtain homology models of proteins which have no known structure. Applications that are enabled include computational studies such as the interactions with ligands or drugs [7-9]. Computational studies to characterize the proteins are thereby supported by PSI. Follow-on experimental studies are supported through the provision of the DNA clones of the determined structures, as available through the Materials Repository [10].

There are a proportionate number of follow-on functional characterization studies that are performed by the broader scientific community after determination of protein structures by the PSI [11]. But there is a latency in these functional characterization studies that reflects the natural pace at which these studies are undertaken and completed. With the entrance of PSI:Biology, there is now more participation of scientists from the biomedical community studying the functions of the structures. This is done in a coordinated manner and it is subject to the NIH peer-review process of partnership formation. The resultant PSI:Biology Partnerships direct structural projects from start to finish with regard to the selection of targets and the concurrent functional characterization studies.

The overall impact of the structural results of PSI for the biomedical community at large is monitored and assessed in part by accounting for the total number of structures determined. A corresponding online metrics site lists the resultant numbers of structures [12]. For the structures determined through PSI:Biology, the user can examine the numbers of structures represented for different phyla and organisms, e.g. eukaryotic, prokaryotic, and human proteins. The metrics site also lists the number of PSI:Biology publications, citations, and total journal impact. These can also be used as measures of the biomedical importance of the research being done in PSI:Biology. These measures account for some of the information that is generated through the PSI. But summaries regarding the specific functions of the determined structures, as assessed by external biomedical resources, are necessary. These external measures provide a further understanding of the focus and depth of the structure to function relationships that have been characterized.

In the current study, we describe the biological relevance of the structures determined through PSI:Biology to estimate the relative impact that the project has for the elucidation of structure to function relationships. A protein here is viewed as having higher biological relevance if it has been studied to a greater extent by the biomedical community. The annotations used in the study are those that represent biological functions that can be attributed to more than one protein. The relative impact of PSI:Biology is quantified using the objective measures of the rates of annotation assignments made by biomedical resources that are external to PSI.

We utilize protein knowledgebases that comprehensively integrate and describe the broad array of biological functions of proteins. The UniProt knowledgebase is used, which characterizes protein sequences on the scales of all known proteomes [13,14]. Also used are the functional annotations obtained through the Structural Biology Knowledgebase (SBKB) from sources external to PSI [6,15]. These annotations and others from open sources described herein provide a perspective regarding the wide range of functions that are attributed to the determined protein structures.

To see the relative impact of PSI:Biology, we examine the average rates at which annotations are assigned to PSI:Biology structures relative to other structural projects. One comparison is for PSI:Biology relative to PSI:1&2. A second is for structures determined through the PSI:Biology Partnerships as compared to structures determined by US authors in which they do not participate in such partnerships.

## Results and discussion

### Comparison of PSI:1&2 to PSI:Biology

**Figure 1:**
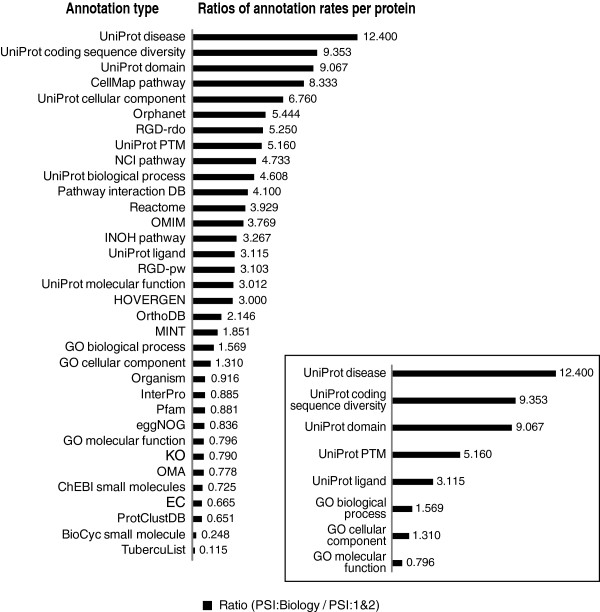
**Ratios of mean number of annotation assignments per protein from PSI:Biology versus PSI:1&2.** A ratio greater than 1 indicates that on average there are more annotation assignments per protein structure from PSI:Biology than from PSI:1&2. Data for the plot is available in Additional file 1: Table S1. Inset- Ratios for a representative groups of annotations based on the UniProt keyword system.

**Figure 2:**
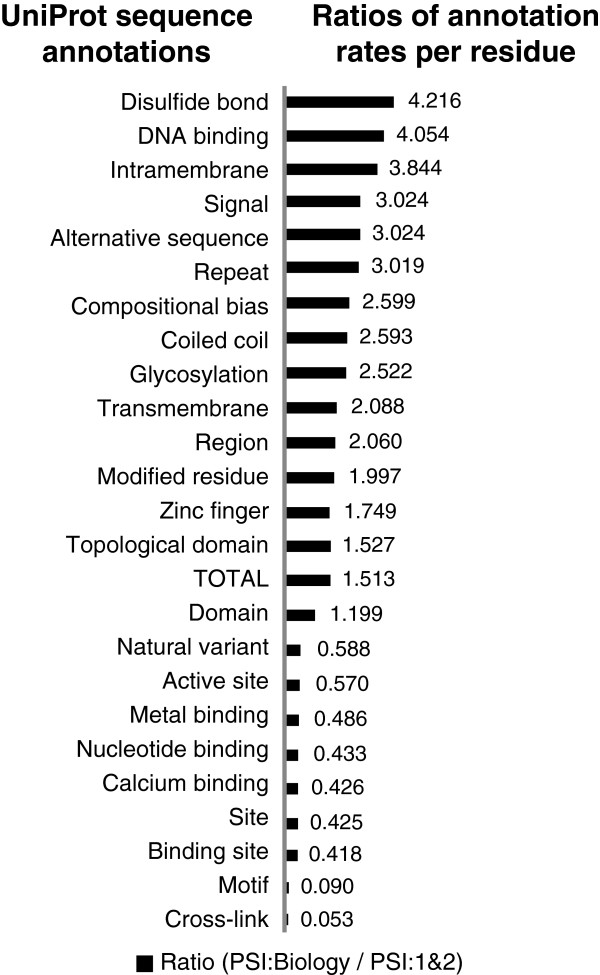
**Ratios of mean number of UniProt annotation assignments per residue from PSI:Biology versus PSI:1&2.** The plot shows the ratios of the mean numbers of UniProt annotation assignments per residue for proteins addressed in all PSI:Biology projects versus all PSI:1&2 projects. Data for the plot is available in Additional file 1: Table S2. A ratio greater than 1 indicates that on average there are more assignments per residue for that annotation type in PSI:Biology as compared to PSI:1&2.

For this comparison, we measure the impact of PSI:Biology relative to PSI:1&2 to see the differences in the trends of the annotation rates of structures. Figure 1 compares the potential impact of PSI:Biology structures with those coming from PSI:1&2. Specifically, we calculated the ratios of the mean number of assignments per structure for a range of annotations from multiple data sources. PSI:Biology structures are more highly annotated across most of the resources that describe functional and phenotypic associations. These results are statistically significant as judged by the Student’s *t*-test (*p*-value ≤ 0.05). For example, PSI:Biology structures are relatively more highly annotated in the Orphanet resource, (*p*-value < 0.001), a resource that describes associations of proteins with rare disease [16]. From the perspective of annotations assigned to particular residues within the structures, we also see that rates of PSI:Biology are higher than PSI:1&2. See Figure 2. **These results support the assertion that PSI:Biology has focused on structure determinations of higher biomedical interest than those examined during PSI:1&2.** See Additional file 1: Tables S1 and S2 for the data used in the plots.

As assessed from the perspective of a broad spectrum of biomedical annotations shown, most annotation assignment rates are higher across PSI:Biology structures relative to PSI:1&2. The types of functions well represented in PSI:Biology tend to be those that are associated with higher order biological processes rather than molecular level functions. For example, we see that Gene Ontology biological processes and UniProt disease keyword annotations are greater for PSI:Biology, but the rate of Gene Ontology molecular functions is lower. We also see a greater percentage of mammalian and human source organism assignments when viewing the organism annotations for PSI:Biology relative to PSI:1&2. We infer that this higher representation of complex biology characterizes PSI:Biology.

The numbers of structures served as a metric for the first two phases of PSI, PSI:1 and PSI:2. It measured the progress toward the goals of these phases which included the development of methods for high-throughput structure determination and the determination protein structures that address different protein sequence families. The metric is applied for PSI:Biology where structures are determined in high throughput using the methods, coordination, and expertise developed during the first two phases. We see that the PSI:Biology numbers of structures metric describes the degree to which high throughput is sustained, but it does not address all the progress towards its goals of determining biomedically relevant structure to function connections. The inference is made based on the result that there are a greater number of annotation rates that are higher in PSI:Biology compared to PSI:1&2. To more fully address the progress, we introduce the annotation metric as a way to more fully capture the numbers of structure to function connections addressed in PSI:Biology. As applied, it is an objective metric since it utilizes annotations of function that are made by biomedical sources that are external to PSI.

The annotation metric assesses a broad range of functions and can be viewed as a complement to the established metric of counting the numbers of structures. Further, it is complementary to another established metric of the impact of PSI that is based on the corresponding publication records of the structures. These are described in the SBKB’s publication portal [6]. We see that the annotation metric can be considered in the context of the established metrics. For example, there are proteins that are biologically relevant that have yet to be studied to great extent by the scientific community, such as those developed through protein design [17]. These proteins will have a low number of annotations yet they will maintain a relatively high biomedical relevance. A more optimal measure of impact for such structures may be based on their corresponding publication records. Also an additional measure of the rate of follow-on studies based on the structure is to be considered. One such measure has been developed to examine PSI-2 structures [11], and may continue to be applied to PSI:Biology structures. We see that the publication records, numbers of structures metric, and annotation metric provide complementary perspectives regarding the impact of PSI:Biology.

### Comparison of PSI:Biology Partnerships to US non-SG ensemble

We next compare structures determined as a result of the PSI:Biology Partnerships to non-structural genomics structures determined by US authors available from the PDB (PDB US non-SG ensemble). There are 117 structures identified as resulting from PSI:Biology Partnerships, and 5613 structures are identified in the US non-SG ensemble. A stated goal in the composition of the PSI:Biology Partnerships is to determine protein structures that address broad, challenging biological questions [1,6]. These projects are subject to the NIH peer-review process. The comparison set is the US non-SG ensemble, which represents all structures determined by US authors exclusive of structural genomics.

**Figure 3:**
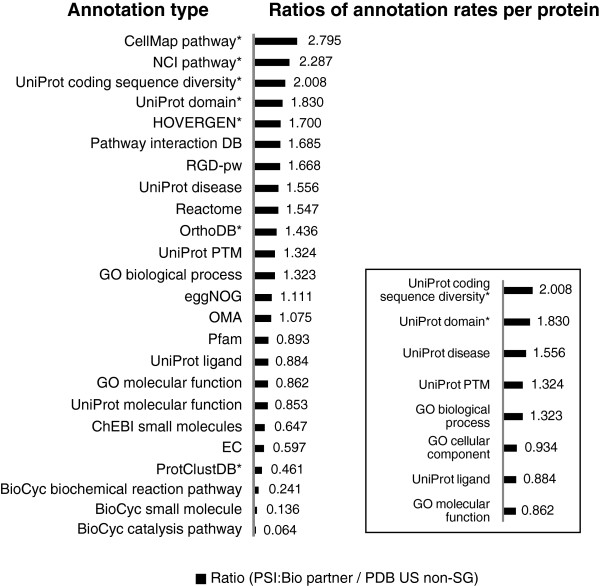
**Ratios of mean number of annotation assignments per protein from PSI:Biology Partnerships versus the PDB US non-SG ensemble.** Asterisks indicate the annotation ratios that are statistically significant based on a Student’s *t*-test (*p*-value ≤ 0.05). Data for the plot is available in Additional file 1: Table S3. Inset- Ratios for a group of annotations based on the UniProt keyword system.

Figure 3 shows that the Partnership structures tend to have higher annotation rates than structures from the PDB US non-SG ensemble. For the comparison, we see that there is a focus on higher order biological processes and diseases. One illustrative example is a higher focus on signaling pathways related to human cancer, as exhibited in the representation of proteins in the National Cancer Institute’s Pathway Interaction Database [18]. A second illustrative example is a greater focus on coding sequence diversity, which includes complex annotations of splice variants [19]. UniProt domains are also higher, which indicates a focus on biologically relevant domains. Noteworthy exceptions are enzymes, annotated with EC numbers, and relevant ligands, as suggested by BioCyc small molecule entries. We interpret this finding as a result of a selection bias against enzymes within the PSI:Biology Partnerships. See Additional file 1: Table S3 for data used in Figure 3.

There is a separate structural genomics initiative that focuses on enzymes [20], which was excluded from the US non-structural genomics PDB ensemble. Thus, there appears to be higher emphasis on biological relevance in PSI:Biology Partnership structures versus US, non-structural genomics structures in the PDB. We believe that most of the US, non-structural genomics structures in the PDB represent structures determined under the auspices of individual investigator funding mechanisms (i.e., primarily R01 funded structure determinations).

For the comparison between PSI:Biology Partnerships and the PDB subset, the mean number of annotation assignments for a representative group of annotation types based on the UniProt keyword system is also examined [13]. See Figure 3, inset. These keywords address different types of functions of proteins and aid in the reduction of redundancy in the data. They also have the quality of being manually reviewed (those with UniProtKB/Swiss-Prot entries) or assigned by rules (those with UniProtKB/Tremble entries) [21]. The UniProt keyword annotations were normalized according to their relative occurrence, and the average of the normalized annotation rates were used to estimate an overall degree to which a protein structure is annotated. This comparison indicates that there are approximately 30% more annotations per protein on average when comparing PSI Biology Partnership structures to the structures from the PDB US non-SG ensemble. Analyses of the average ratios and the normalized ratios indicate that they are statistically different between these groups. See Table 1.

**Table 1:** Mean numbers of annotation assignments per protein for eight representative annotation types

				**PDB**	**PSI:Biology**
	**PDB**	**PSI:Biology**		**US non-SG**	**Partnerships**
	**US non-SG**	**Partnerships**	**Ratio of**	**Normalized**	**Normalized**
**Annotation type**	**Means**	**Means**	**Means**	**Means**	**Means**
GO biological process	4.385	5.802	1.323	1.359	1.798
GO cellular component	1.889	1.765	0.934	1.327	1.239
GO molecular function	1.961	1.691	0.862	1.149	0.991
UniProt coding sequence diversity	0.369	0.741	2.008	1.299	2.609
UniProt disease	0.151	0.235	1.556	1.411	2.196
UniProt domain	0.695	1.272	1.830	1.271	2.325
UniProt ligand	1.006	0.889	0.884	1.168	1.033
UniProt PTM	1.343	1.778	1.324	2.050	2.715
**Mean**			**1.340**	**1.379**	**1.863**
**std. err.**			**0.154**	**0.101**	**0.248**
***p*-value**			**0.032**		**0.052**

The mean numbers of annotation assignments for the PDB US non-SG ensemble and the structures determined by PSI:Biology Partnerships are shown in the first two columns respectively. The third column shows the ratios of these means. The average of the eight ratios is calculated for an overall mean ratio. A 1-tailed unpaired *t*-test is performed to test the null hypothesis that the overall mean ratio is greater than 1 (*p*-value = 0.0317). In the fourth and fifth data columns, the normalized means are shown, where the normalization is done by dividing by the rates of annotation assignments by their corresponding means for the entire PDB for structures deposited during the relevant time frame (July 1, 2010 through February 28, 2013). A 1-tailed unpaired *t*-test is performed on the data sets to test the null hypothesis that the average of the means for PSI:Biology Partnership structures is greater than that for the PDB US non-SG ensemble (*p*-value = 0.0517).

**Figure 4:**
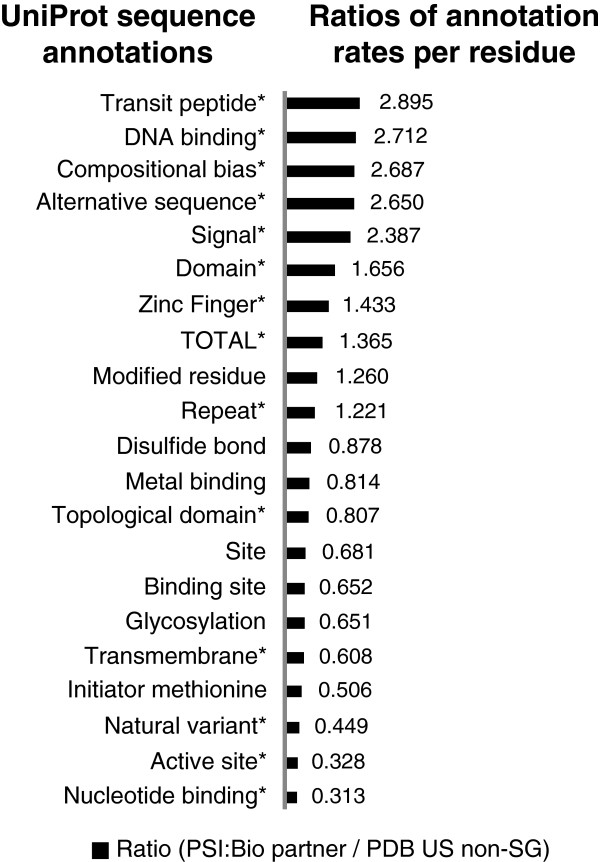
**Ratios of rates of UniProt annotation assignments per residue for PSI:Biology Partnerships structures versus non-structural genomics structures in the PDB.** Asterisks indicate the rates that are statistically different between the sets based on a Student’s *t*-test (*p*-value ≤ 0.05). A ratio greater than 1 indicates that on average there are more assignments per residue for that annotation in structures from PSI:Biology Partnerships than from PDB US non-SG structures. Data for the plot is available in Additional file 1: Table S4.

We also see that the rates of the residue annotation assignments based on the UniProt system further support the finding that there is approximately a 1.3 times higher assignment rate for PSI:Biology Partnership structures relative to the PDB US non-SG ensemble. The residue assignments are based on UniProt’s system of identifying sequence features. We view these residue level assignments as addressing different functions, and we see that they can be accumulated at the same scale to give an overall assignment rate per residue for each data set. See Figure 4 with the supplementary data provided in Additional file 1: Table S4. As a further test, we excluded the annotation types that we regarded as largely computational derived, e.g. Compositional bias, Transmembrane, Coiled coil, Domain, Repeat, Signal, and Zinc finger. The results show that annotation rates for the residue level assignments for PSI:Biology Partnerships are higher than for the PDB US non-SG ensemble, *p*-value = 2.9×10^-30^.

The highest rate difference for the residue level assignments was for Transit peptide. That makes sense given that there is a Mitochondrial Protein Partnership that determines structures of associated with mitochondrion [1,22]. Also prevalent are proteins that are alternatively spliced. Such proteins are seen as a relative focus given their importance in protein disorder [23], which is relevant to PSI:Biology projects that involve signaling networks [24]. We see that further review of relative prevalence of the residue level annotations across the range of highly specific functions further reveals details of the relative focus of the PSI:Biology Partnerships.

## Conclusions

There are two major findings from these analyses. First, PSI:Biology indeed has a higher focus on proteins documented as being biomedically relevant than PSI:1&2. This finding validates the annotation rate metric is a means to measure the impact of PSI:Biology with regard to the numbers of structure to biological function connections examined. The metric is complementary to established metrics of the numbers of structures and the corresponding publication record of the structures. Second, there is clear evidence that PSI:Biology Partnerships have determined structures that are found to have a higher biomedical relevance than that found for US generated, non-structural genomics structures. As more structures are determined through PSI:Biology Partnerships and more data is evaluated, we expect this trend to become more apparent based on the many annotation types analyzed.

An inference of the second finding is that PSI:Biology Partnerships provide an added value towards the goals of PSI:Biology with regard to the resultant biological relevance of the determined structures. **We interpret the result as evidence that PSI:Biology Partnerships are focusing on the determination of protein structures for which there is a higher interest to the biomedical community as judged through the representative annotation types.** These representative annotations are based on the UniProt keyword system and based on the system developed by UniProt to identify different residue level assignments of function. Through the elucidation of structures, we see the team-based PSI:Biology Partnerships as tying together the varied functional information of proteins that have high biomedical relevance.

We assess that the added value of the PSI:Biology Partnerships is something that has not previously been quantified, and represents some of the progress toward the goals of PSI:Biology. The NIH’s implementation of peer-reviewed structural projects that involve the formation of well established collaborations between structural genomics centers and biomedical scientists in the community are seen as providing a viable means to achieve more valuable structures. We see that the annotation metric can be used to monitor the progress towards the goals of PSI:Biology. In particular we see the metric as a means to quantify the added value and benefits of the synergies realized through PSI:Biology Partnerships.

To facilitate access to the results of the PSI:Biology projects based on the annotation rates, a website with interactive charting is available [25]. The site describes the datasets used in the study in interactive form, and it provides access to current updates of the annotations across all structures in the PDB. A corresponding weekly updated analysis of the annotation rates of PSI:Biology structures is available.

## Methods

### Annotation assembly

The first step in the data assembly was to review biomedical databases that annotate proteins and decide which annotations therein to use in the study. All resources were required to have an open-source policy of freely available information to the scientific community. We identified assignments of annotations that can pertain to more than one protein such that none of the assignments used were specific to a single protein. Different proteins are defined as having a different primary accession code in UniProt. Annotations from a specific resource or subcategory of a specific resource were grouped as annotation types. A list of 43 annotation types that were identified is given in Additional file 1: Table S5.

Assignments of annotations were mapped to protein structures based on UniProt accession code correspondences for each protein structural chain. Information provided through UniProt data files and the SBKB were utilized. In addition, information was directly extracted from BioCyc [26], HumanCyc, NCI pathway [18], INOH pathway [27], CellMap pathway, ChEBI ligand [28], RGD [29], MGI [30], and OMIM [31]. We used from RGD disease ontology (rdo) and pathway (pw) information for rat, human, and mouse. The MGI database was used to obtain mammalian phenotype information for mouse proteins. Phenotype information was used from OMIM.

As a representative group of annotation types, we used eight UniProt keyword categories. These keywords were viewed as covering a wide variety of annotations while remaining relatively independent with respect to the information that they provide. The UniProt keyword category called developmental stage was not used due to the use of non-standardized text descriptions instead of keyword assignments in some cases, and due to the relative scarcity of these annotations. The keywords of technical terms were not used as these can refer to the experimental system in which the protein was studied rather than a functional attribute of a protein.

A refinement of the keyword system for our purpose was to substitute the corresponding GO terms for the three keyword categories in UniProt– biological process, molecular function, and cellular component. The UniProt keyword entries are made to be different and complementary to GO terms in many cases, but there are significant synonymous cross-references as reflected in the mapping files. The choice to use GO terms was primarily based on the fact that not only are GO terms annotated manually and electronically by a specialized group of UniProt curators, but also manually by approximately 36 supplemental external groups as part of the GO Consortium [32]. We also see that GO annotations in UniProt are generally limited to specific terms at the lowest leaves of the GO hierarchy. This has been verified by UniProt staff through a personal communication. We rely also on mappings between GO terms and structures from the SIFTs project [33], which adds another layer of review for these annotations.

Assignments of annotations of residues were taken from sequence annotation features as provided through UniProt. For the purposes here, only those annotation assignments that described as naturally occurring functions were used. In total, 29 types of annotation assignments from the group sequence annotation features were obtained. A list of the types of annotations used can be found in Additional file 1: Table S6.

The residue specific annotations from UniProt were mapped to those residues that were found within the region with a determined structure, and annotations outside these regions were not considered further. Mapping was done by using the information in the UniProt flat files that describe the functions of each residue. The information on the residue number correspondences between the UniProt sequence files and the PDB coordinate files were provided through the SIFTs project [33]. We used the mapping files see which annotations fell within the region of the protein with a determined structure.

### Data sets

Protein structures deposited to the PDB were separated into three discrete data sets for comparison – PSI:1&2, PSI:Biology, and PDB US non-SG. The PSI:1&2 set (5138 structures in total) combined all structures determined during the first 2 phases of the PSI. PSI:Biology (1017 structures in total) contained all structures considered part of the third phase of the PSI. Any structures from the PSI projects that had multiple projects attributed to them, for example due to being studied at different structural genomics centers, were assigned to their earliest associated project. This was done to eliminate mislabeling protein structures that were determined in either PSI:1 or PSI:2 but subsequently have had a functional assay performed on them during PSI:Biology.

The third data set, PDB US non-SG (5613 structures), was comprised of US-only structures in the PDB deposited during the PSI:Biology time period, excluding those determined by structural genomics programs. We defined a PSI:Biology time period as structures deposited to the PDB from July 1, 2010 through February 28, 2013. Structures in the PSI:Biology project were separated into subsets based on their target categories as described in the TargetTrack database [34,35]. PSI:Biology Partnership structures were chosen as the subset of PSI:Biology for a comparison to the PDB US non-SG data set. Of the 1017 structures determined in PSI:Biology as a whole, there were 117 protein structures identified as resulting from PSI:Biology Partnerships.

### Data analyses

Each protein structure was associated with a UniProt accession code using information from SIFTS (5). Chimeric proteins (those protein structures that are associated with more than one UniProt accession) were removed from the data sets. The number of unique UniProt accession codes for each data set was 4444, 790, 81, and 2624 for PSI:1&2, PSI:Biology, PSI:Biology Partnerships, and PDB US non-SG respectively.

The rate, or mean numbers, of annotations per protein structure for each data set were calculated. The sum of the number of unique annotations was divided by the number of associated unique UniProt accession codes. For the residue level annotation analyses, the total number of residues for each project was split into groups of 250 residues, which was the average length of all the structures studied. There were 3842 groups for PSI:1&2, 815 groups for PSI:Biology, 62 groups for PSI:Biology Partnerships, and 2947 groups for the PDB US non-SG ensemble. The rates per residue of each sequence feature for residue level annotations were calculated by dividing the number of instances found per each group of 250 residues by 250. The average across all the groups of 250 for each project was obtained.

The rates of annotation for the protein level assignments and the residue level assignments were compared between data sets. Student’s *t*-tests were conducted to show which annotation types had significantly different values between the data sets. For the comparison between the set of PSI:Biology Partnership structures to the set of PDB US non-SG structures, the average of the mean ratios of the annotation rates at the protein level across the representative group of annotation types based on the UniProt keyword system were tested to be greater than 1. The t-statistic was calculated by *t = (x’ – 1) / SE* where *x’* is the average of the means and *SE* is the associated standard error. The mean numbers of annotations per protein for the representative annotation types (UniProt keywords combined with GO terms as described above) were also normalized to the mean for the given category as obtained for the entire PDB over the time frame of PSI:Biology. That time frame was July 1, 2010 to February 28, 2013. A 1-tailed unpaired *t*-test with the null hypothesis that the average normalized values across the annotation types for the two groups of structures was conducted.

## Abbreviations

PSI:Biology: the Protein Structure Initiative:Biology program; PSI:1&2: the first two phases of the Protein Structure Initiative; PDB: Protein Data Bank; PDB US non-SG ensemble: protein structures determined from July 1, 2010 through February 28, 2013 by US authors with structural genomics structures excluded; UniProt: Universal Protein Resource; PDB: Protein Data Bank; SBKB: Structural Biology Knowledgebase; CellMap: The Cancer Cell Map; INOH: Integrated Network Objects with Hierarchies; NCI: National Cancer Institute; DrugBank: Open Data Drug and Drug, Target Database; ChEBI: Chemical Entities of Biological Interest at the European Bioinformatics Institute; OMIM: Online Mendelian Inheritance in Man; GO: Gene Ontology; EC: Enzyme Commission; Pfam: Protein family database; RGD-rdo: the RGD disease ontology in the Rat Genome Database; PTM: Post-translational modification; PW: Pathway; MGI: Mouse Genome Informatics; KO: Kyoto Encyclopedia of Genes and Genomes object; OMA: A comprehensive, automated project for the identification of orthologs from complete genome data; ProtClusDB: National Center Biotechnology Information clusters of proteins with similar sequences.

## Competing interests

The authors declare that they have no competing interests.

## Authors’ contributions

WM identified the annotations that were utilized. WM, PD, and EJ created the programs for the integration of annotations and performed the data analysis. WM conceived the project. PD and WM wrote the paper. All authors read and approved the final manuscript.

## Supplementary Material

Additional file 1: Table S1 Mean number of annotations per PSI:Biology and PSI:1&2 proteins across varied biomedical resources. **Table S2.** Mean number of UniProt sequence annotations per residue for PSI:Biology and PSI:1&2 structures. **Table S3.** Mean number of annotations per PSI:Biology Partnership protein and per PDB US non-SG protein across resources. **Table S4.** Mean number of UniProt sequence annotations per residue for PSI:Biology Partnership and PDB US non-SG structures. **Table S5.** List of the 43 annotation types used in the analysis. **Table S6.** List of the 29 sequence annotations used in the residue level analysis. **Table S7.** Mean number of annotations per protein for eight UniProt keyword annotation types. Click here for file
