# Supplementary material for: Quantification of the impact of PSI:Biology according to the annotations of the determined structures
Source: BMC Struct Biol. 2013 Oct 21;13:24. doi: 10.1186/1472-6807-13-24 (PMC4016320; doi:10.1186/1472-6807-13-24)
Supplement: Additional file 1: Table S1 — Mean number of annotations per PSI:Biology and PSI:1&2 proteins across varied biomedical resources. Table S2. Mean number of UniProt sequence annotations per residue for PSI:Biology and PSI:1&2 structures. Table S3. Mean number of annotations per PSI:Biology Partnership protein and per PDB US non-SG protein across resources. Table S4. Mean number of UniProt sequence annotations per residue for PSI:Biology Partnership and PDB US non-SG structures. Table S5. List of the 43 annotation types used in the analysis. Table S6. List of the 29 sequence annotations used in the residue level analysis. Table S7. Mean number of annotations per protein for eight UniProt keyword annotation types. [file 1472-6807-13-24-S1.docx]

**Additional file 1**

**Table S1: Mean number of annotations per PSI:Biology and PSI:1&2 proteins across varied biomedical resources.**

|  |  | **PSI:1&2** | | **PSI:Biology** | |  |
| --- | --- | --- | --- | --- | --- | --- |
|  |  | **Mean #** |  | **Mean #** |  |  |
|  | **Ratio** | **annotations** | **standard** | **annotations** | **standard** |  |
| **Annotation type** | **(bio/pdb)** | **per protein** | **error** | **per protein** | **error** | **p-value** |
| UniProt disease | 12.400 | 0.005 | 0.001 | 0.062 | 0.014 | < 0.001 |
| UniProt coding sequence diversity | 9.353 | 0.017 | 0.002 | 0.159 | 0.018 | < 0.001 |
| UniProt domain | 9.067 | 0.030 | 0.004 | 0.272 | 0.028 | < 0.001 |
| CellMap pathway | 8.333 | 0.003 | 0.001 | 0.025 | 0.008 | < 0.001 |
| UniProt cellular component | 6.760 | 0.050 | 0.005 | 0.338 | 0.031 | < 0.001 |
| Orphanet | 5.444 | 0.009 | 0.002 | 0.049 | 0.014 | < 0.001 |
| RGD-rdo | 5.250 | 0.008 | 0.005 | 0.042 | 0.027 | 0.047 |
| UniProt PTM | 5.160 | 0.075 | 0.008 | 0.387 | 0.043 | < 0.001 |
| NCI pathway | 4.733 | 0.030 | 0.011 | 0.142 | 0.042 | < 0.001 |
| UniProt biological process | 4.608 | 0.074 | 0.007 | 0.341 | 0.033 | < 0.001 |
| Pathway interaction DB | 4.100 | 0.020 | 0.008 | 0.082 | 0.029 | 0.004 |
| Reactome | 3.929 | 0.028 | 0.004 | 0.110 | 0.023 | < 0.001 |
| OMIM | 3.769 | 0.013 | 0.003 | 0.049 | 0.014 | < 0.001 |
| INOH pathway | 3.267 | 0.015 | 0.005 | 0.049 | 0.024 | 0.027 |
| UniProt ligand | 3.115 | 0.087 | 0.007 | 0.271 | 0.029 | < 0.001 |
| RGD-pw | 3.103 | 0.058 | 0.013 | 0.180 | 0.053 | 0.001 |
| UniProt molecular function | 3.012 | 0.084 | 0.007 | 0.253 | 0.025 | < 0.001 |
| HOVERGEN | 3.000 | 0.041 | 0.003 | 0.123 | 0.012 | < 0.001 |
| OrthoDB | 2.146 | 0.048 | 0.003 | 0.103 | 0.011 | < 0.001 |
| MINT | 1.851 | 0.047 | 0.003 | 0.087 | 0.010 | < 0.001 |
| GO biological process | 1.569 | 0.914 | 0.037 | 1.434 | 0.197 | < 0.001 |
| GO cellular component | 1.310 | 0.365 | 0.014 | 0.478 | 0.046 | 0.003 |
| Organism | 0.916 | 0.909 | 0.004 | 0.833 | 0.014 | < 0.001 |
| InterPro | 0.885 | 2.297 | 0.028 | 2.033 | 0.069 | < 0.001 |
| Pfam | 0.881 | 1.022 | 0.012 | 0.900 | 0.029 | < 0.001 |
| eggNOG | 0.836 | 0.549 | 0.007 | 0.459 | 0.018 | < 0.001 |
| GO molecular function | 0.796 | 1.121 | 0.018 | 0.892 | 0.045 | < 0.001 |
| KO | 0.790 | 0.423 | 0.008 | 0.334 | 0.017 | < 0.001 |
| OMA | 0.778 | 0.803 | 0.006 | 0.625 | 0.017 | < 0.001 |
| ChEBI ligand | 0.725 | 3.541 | 0.139 | 2.566 | 0.145 | 0.005 |
| EC | 0.665 | 0.227 | 0.007 | 0.151 | 0.013 | < 0.001 |
| ProtClustDB | 0.651 | 0.737 | 0.007 | 0.480 | 0.018 | < 0.001 |
| BioCyc small molecule | 0.248 | 0.286 | 0.032 | 0.071 | 0.029 | 0.005 |
| TubercuList | 0.115 | 0.026 | 0.002 | 0.003 | 0.002 | < 0.001 |

The *p*-values of Student’s *t*-tests to compare the means for the PSI:Biology versus PSI:1&2 are given on the rightmost column.

**Table S2: Mean number of UniProt sequence annotations per residue for PSI:Biology and PSI:1&2 structures.**

|  |  | **PSI:1&2** | | **PSI:Biology** | |  |
| --- | --- | --- | --- | --- | --- | --- |
|  |  | **Mean #** |  | **Mean #** |  |  |
| **UniProt sequence** | **Ratio** | **annotations** | **standard** | **Annotations** | **standard** |  |
| **annotations** | **(bio/pdb)** | **per residue** | **error** | **per residue** | **error** | **p-value** |
| Disulfide bond | 4.216 | 0.000217 | 0.000015 | 0.000913 | 0.000067 | < 0.001 |
| DNA binding | 4.054 | 0.000662 | 0.000026 | 0.002680 | 0.000114 | < 0.001 |
| Intramembrane | 3.844 | 0.000175 | 0.000013 | 0.000672 | 0.000059 | < 0.001 |
| Signal | 3.024 | 0.000346 | 0.000019 | 0.001050 | 0.000072 | < 0.001 |
| Alternative sequence | 3.024 | 0.006000 | 0.000094 | 0.018100 | 0.000456 | < 0.001 |
| Repeat | 3.019 | 0.002120 | 0.000047 | 0.006410 | 0.000175 | < 0.001 |
| Compositional bias | 2.599 | 0.000244 | 0.000016 | 0.000633 | 0.000055 | < 0.001 |
| Coiled coil | 2.593 | 0.000312 | 0.000018 | 0.000810 | 0.000062 | < 0.001 |
| Glycosylation | 2.522 | 0.000045 | 0.000007 | 0.000113 | 0.000023 | < 0.001 |
| Transmembrane | 2.088 | 0.003480 | 0.000059 | 0.007260 | 0.000190 | < 0.001 |
| Region | 2.060 | 0.009790 | 0.000105 | 0.020200 | 0.000368 | < 0.001 |
| Modified residue | 1.997 | 0.000265 | 0.000017 | 0.000530 | 0.000053 | < 0.001 |
| TOTAL (non-comp)^†^ | 1.703 | 0.036424 | 0.000219 | 0.062047 | 0.000724 | < 0.001 |
| Zinc finger | 1.749 | 0.000545 | 0.000024 | 0.000952 | 0.000069 | < 0.001 |
| Topological domain | 1.527 | 0.009440 | 0.000098 | 0.014400 | 0.000266 | < 0.001 |
| TOTAL | 1.513 | 0.086100 | 0.000341 | 0.130000 | 0.001020 | < 0.001 |
| Domain | 1.199 | 0.042600 | 0.000206 | 0.051100 | 0.000476 | < 0.001 |
| Natural variant | 0.588 | 0.000710 | 0.000036 | 0.000417 | 0.000048 | < 0.001 |
| Active site | 0.570 | 0.000422 | 0.000021 | 0.000240 | 0.000033 | < 0.001 |
| Metal binding | 0.486 | 0.002640 | 0.000057 | 0.001280 | 0.000083 | < 0.001 |
| Nucleotide binding | 0.433 | 0.003340 | 0.000060 | 0.001440 | 0.000086 | < 0.001 |
| Calcium binding | 0.426 | 0.000300 | 0.000018 | 0.000128 | 0.000026 | < 0.001 |
| Site | 0.425 | 0.000138 | 0.000012 | 0.000059 | 0.000017 | 0.003 |
| Binding site | 0.418 | 0.001570 | 0.000041 | 0.000658 | 0.000055 | < 0.001 |
| Motif | 0.090 | 0.000437 | 0.000021 | 0.000039 | 0.000014 | < 0.001 |
| Cross-link | 0.053 | 0.000093 | 0.000010 | 0.000005 | 0.000005 | < 0.001 |
| Propeptide | 0.000 | 0.000048 | 0.000016 | 0.000000 | 0.000000 | < 0.001 |
| Peptide | 0.000 | 0.000032 | 0.000060 | 0.000000 | 0.000000 | < 0.001 |

The *p*-values of Student’s *t*-tests to compare the means for PSI:Biology versus PSI:1&2 are given on the rightmost column. The ratio of the total number of all sequence annotations per residue is 1.513 (p-value < 0.001). Note the last two rows have a ratio of zero because no residues in PSI:Biology had those features.^†^ TOTAL (non-comp) excludes the following sequence annotations, which are estimated to be largely computationally derived: Signal, Zinc finger, Compositional bias, Transmembrane, Coiled coil, Domain, and Repeat.

**Table S3: Mean number of annotations per PSI:Biology Partnership protein and per PDB US non-SG protein across resources.**

|  |  | **PDB US non-SG** | | **PSI:Biology Partnership** | |  |
| --- | --- | --- | --- | --- | --- | --- |
|  |  | **Mean #** |  | **Mean #** |  |  |
|  | **Ratio** | **annotations** | **standard** | **annotations** | **standard** |  |
| **Annotation type** | **(bio/pdb)** | **per protein** | **error** | **per protein** | **error** | **p-value** |
| CellMap Pathway* | 2.795 | 0.044 | 0.006 | 0.123 | 0.054 | 0.018 |
| NCI Pathway* | 2.287 | 0.432 | 0.049 | 0.988 | 0.371 | 0.051 |
| UniProt Coding sequence diversity* | 2.008 | 0.369 | 0.013 | 0.741 | 0.097 | < 0.001 |
| UniProt Domain* | 1.830 | 0.695 | 0.023 | 1.272 | 0.172 | < 0.001 |
| HOVERGEN* | 1.700 | 0.327 | 0.009 | 0.556 | 0.056 | < 0.001 |
| Pathway_Interaction_DB | 1.685 | 0.330 | 0.039 | 0.556 | 0.267 | 0.319 |
| RGD-pw | 1.668 | 0.659 | 0.056 | 1.099 | 0.381 | 0.179 |
| UniProt Disease | 1.556 | 0.151 | 0.012 | 0.235 | 0.069 | 0.212 |
| Reactome | 1.547 | 0.351 | 0.022 | 0.543 | 0.171 | 0.128 |
| OrthoDB* | 1.436 | 0.335 | 0.009 | 0.481 | 0.056 | 0.008 |
| UniProt PTM | 1.324 | 1.343 | 0.046 | 1.778 | 0.248 | 0.101 |
| GO Biological process | 1.323 | 4.385 | 0.170 | 5.802 | 1.441 | 0.157 |
| eggNOG | 1.111 | 0.611 | 0.010 | 0.679 | 0.052 | 0.233 |
| OMA | 1.075 | 0.574 | 0.010 | 0.617 | 0.054 | 0.451 |
| Pfam | 0.893 | 1.451 | 0.029 | 1.296 | 0.131 | 0.357 |
| UniProt Ligand | 0.884 | 1.006 | 0.028 | 0.889 | 0.136 | 0.463 |
| GO Molecular function | 0.862 | 1.961 | 0.041 | 1.691 | 0.185 | 0.256 |
| UniProt Molecular function | 0.853 | 1.042 | 0.027 | 0.889 | 0.128 | 0.326 |
| ChEBI Ligand | 0.647 | 7.330 | 0.250 | 4.741 | 0.838 | 0.070 |
| EC | 0.597 | 0.352 | 0.013 | 0.210 | 0.049 | 0.066 |
| ProtClustDB* | 0.461 | 0.295 | 0.009 | 0.136 | 0.038 | 0.002 |
| BioCyc Biochemical Reaction Pathway | 0.241 | 0.203 | 0.026 | 0.049 | 0.039 | 0.302 |
| BioCyc Small Molecule | 0.136 | 0.456 | 0.050 | 0.062 | 0.062 | 0.167 |
| BioCyc Catalysis Pathway | 0.064 | 0.188 | 0.025 | 0.012 | 0.012 | 0.224 |

The *p*-values of Student’s *t*-tests to compare the means are given on the right-most column.

**Table S4: Mean number of UniProt sequence annotations per residue for PSI:Biology Partnership and PDB US non-SG structures.**

|  |  | **PDB US non-SG** | | **PSI:Biology Partnership** | |  |
| --- | --- | --- | --- | --- | --- | --- |
|  |  | **Mean #** |  | **Mean #** |  |  |
| **UniProt sequence** | **Ratio** | **annotations** | **standard** | **annotations** | **standard** |  |
| **annotations** | **(bio/pdb)** | **per residue** | **error** | **per residue** | **error** | **p-value** |
| Transit peptide* | 2.895 | 0.000267 | 0.000019 | 0.000774 | 0.000258 | < 0.001 |
| DNA binding* | 2.712 | 0.003070 | 0.000065 | 0.008320 | 0.000732 | < 0.001 |
| Compositional bias* | 2.687 | 0.003000 | 0.000063 | 0.008060 | 0.000744 | < 0.001 |
| Alternative sequence* | 2.650 | 0.049200 | 0.000335 | 0.130000 | 0.004070 | < 0.001 |
| Signal* | 2.387 | 0.000622 | 0.000030 | 0.001480 | 0.000335 | < 0.001 |
| Domain* | 1.656 | 0.151000 | 0.000414 | 0.250000 | 0.003020 | < 0.001 |
| Zinc finger* | 1.433 | 0.002520 | 0.000057 | 0.003610 | 0.000466 | 0.007 |
| TOTAL* | 1.365 | 0.443000 | 0.000882 | 0.605000 | 0.006720 | < 0.001 |
| Modified residue | 1.260 | 0.002050 | 0.000061 | 0.002580 | 0.000432 | 0.211 |
| TOTAL (non-comp)^†^ | 1.223 | 0.249120 | 0.000687 | 0.304645 | 0.005676 | < 0.001 |
| Repeat* | 1.221 | 0.022400 | 0.000168 | 0.027400 | 0.001200 | < 0.001 |
| Disulfide bond | 0.878 | 0.002650 | 0.000061 | 0.002320 | 0.000351 | 0.441 |
| Metal binding | 0.814 | 0.002540 | 0.000065 | 0.002060 | 0.000439 | 0.299 |
| Topological domain* | 0.807 | 0.086200 | 0.000328 | 0.069500 | 0.002020 | < 0.001 |
| Site | 0.681 | 0.000569 | 0.000028 | 0.000387 | 0.000151 | 0.342 |
| Binding site | 0.652 | 0.001780 | 0.000051 | 0.001160 | 0.000324 | 0.080 |
| Glycosylation | 0.651 | 0.001090 | 0.000038 | 0.000710 | 0.000252 | 0.147 |
| Transmembrane* | 0.608 | 0.011100 | 0.000124 | 0.006770 | 0.000745 | < 0.001 |
| Initiator methionine | 0.506 | 0.000255 | 0.000018 | 0.000129 | 0.000091 | 0.318 |
| Natural variant* | 0.449 | 0.006610 | 0.000114 | 0.002970 | 0.000421 | < 0.001 |
| Active site* | 0.328 | 0.000787 | 0.000033 | 0.000258 | 0.000126 | 0.022 |
| Nucleotide binding* | 0.313 | 0.005970 | 0.000092 | 0.001870 | 0.000328 | < 0.001 |
| Motif | 0.000 | 0.001870 | 0.000061 | 0.000000 | 0.000000 | < 0.001 |
| Peptide | 0.000 | 0.001060 | 0.000092 | 0.000000 | 0.000000 | < 0.001 |
| Calcium binding | 0.000 | 0.001010 | 0.000038 | 0.000000 | 0.000000 | < 0.001 |
| Propeptide | 0.000 | 0.000559 | 0.000063 | 0.000000 | 0.000000 | < 0.001 |
| Intramembrane | 0.000 | 0.000537 | 0.000072 | 0.000000 | 0.000000 | < 0.001 |
| Cross-link | 0.000 | 0.000289 | 0.000061 | 0.000000 | 0.000000 | < 0.001 |
| Lipidation | 0.000 | 0.000064 | 0.000092 | 0.000000 | 0.000000 | < 0.001 |

The *p*-values of Student’s *t*-tests to compare the means for PSI:Biology Partnerships versus PDB US non-SG are given on the rightmost column. The ratio of the total number of all sequence annotations per residue is 1.365 (p-value < 0.001). Note the last seven rows have a ratio of zero because no residues in PSI:Biology had those features. ^†^ TOTAL (non-comp) excludes the following sequence annotations, which are estimated to be largely computationally derived: Signal, Zinc finger, Compositional bias, Transmembrane, Coiled coil, Domain, and Repeat.

**Table S5: List of the 43 annotation types used in the analysis**.

| BioCyc biochemical reaction pathway | NCI Pathway Interaction Database |
| --- | --- |
| BioCyc catalysis pathway | OMA[[1](#_ENREF_1)] |
| BioCyc small molecule | OMIM |
| CAZy[[2](#_ENREF_2)] | Organism |
| CellMap pathway | Orphanet |
| ChEBI small molecules | OrthoDB[[3](#_ENREF_3)] |
| DrugBank[[4](#_ENREF_4)] | Pathway interaction DB |
| EC[[5](#_ENREF_5)] | Pfam[[6](#_ENREF_6)] |
| eggNOG[[7](#_ENREF_7)] | ProtClustDB[[8](#_ENREF_8)] |
| **GO biological process** | Reactome[[9](#_ENREF_9)] |
| **GO cellular component** | RGD-pw |
| **GO molecular function** | RGD-rdo |
| HOVERGEN[[10](#_ENREF_10)] | TCDB[[11](#_ENREF_11)] |
| HumanCyc pathway | TubercuList[[12](#_ENREF_12)] |
| HumanCyc small molecule | UniProt biological process |
| HumanCyc biochemical reaction | UniProt cellular component |
| INOH pathway | **UniProt coding sequence diversity** |
| InterPro[[13](#_ENREF_13)] | **UniProt disease** |
| KO[[14](#_ENREF_14)] | **UniProt domain** |
| MGI | **UniProt ligand** |
| MINT[[15](#_ENREF_15)] | UniProt molecular function |
|  | **UniProt PTM** |
|  |  |

Bold denotes the set of eight representative annotation types that are used to compare projects.

**Table S6: List of the 29 sequence annotations used in the residue level analysis**.

| Active site | Modified residue |
| --- | --- |
| Alternative sequence | Motif |
| Binding site | Natural variant |
| Calcium binding | Nucleotide binding |
| Coiled coil | Peptide |
| Compositional bias | Propeptide |
| Cross-link | Region |
| Disulfide bond | Repeat |
| DNA binding | Signal |
| Domain | Site |
| Glycosylation | Topological domain |
| Initiator methionine | Transit peptide |
| Intramembrane | Transmembrane |
| Lipidation | Zinc finger |
| Metal binding |  |

**Table S7:** **Mean number of annotations per protein for eight UniProt keyword annotation types.**

|  |  |  |  | **PDB** | **PSI:Biology** |
| --- | --- | --- | --- | --- | --- |
|  | **PDB** | **PSI:Biology** |  | **US non-SG** | **partnership** |
|  | **US non-SG** | **partnership** | **Ratio of** | **Normalized** | **Normalized** |
| **Annotation type** | **Means** | **Means** | **Means** | **Means** | **Means** |
| UniProt Biological process | 1.183 | 1.111 | 0.939 | 1.256 | 1.179 |
| UniProt Cellular component | 1.254 | 1.272 | 1.014 | 1.305 | 1.324 |
| UniProt Coding sequence diversity | 0.369 | 0.741 | 2.008 | 1.299 | 2.609 |
| UniProt Disease | 0.151 | 0.235 | 1.556 | 1.411 | 2.196 |
| UniProt Domain | 0.695 | 1.272 | 1.830 | 1.271 | 2.325 |
| UniProt Ligand | 1.006 | 0.889 | 0.884 | 1.168 | 1.033 |
| UniProt Molecular function | 1.042 | 0.889 | 0.853 | 1.085 | 0.926 |
| UniProt PTM | 1.343 | 1.778 | 1.324 | 2.050 | 2.715 |
| **Mean** |  |  | 1.301 | 1.356 | 1.788 |
| **std. err.** |  |  | 0.160 | 0.105 | 0.263 |
| ***p*-value** |  |  | **0.051** |  | **0.081** |

The means of the number of annotations for the US non-SG ensemble structures and the structures the results for the PSI:Biology Partnerships are shown in the first two columns respectively. The third column shows the ratios of these means. The average of the eight ratios is calculated for an overall mean ratio. A 1-tailed unpaired *t*-test is performed to test the null hypothesis that the overall mean ratio is greater than 1 (*p*-value = 0.0509). In the fourth and fifth data columns, the normalized means are shown, where the normalization is done by dividing by the rate of annotation of the annotation type by the corresponding mean for the entire PDB of structures deposited during the relevant time frame (July 1, 2010 – February 28, 2013). A 1-tailed unpaired *t*-test is performed on the data sets to test the null hypothesis that the average of the means for PSI:Biology Partnership proteins is greater than that for the PDB US non-SG ensemble (*p*-value = 0.0805).

**Supplementary References**

1. Schneider A, Dessimoz C, Gonnet GH: **OMA Browser—exploring orthologous relations across 352 complete genomes**. *Bioinformatics* 2007, **23**(16):2180-2182.

2. Cantarel BL, Coutinho PM, Rancurel C, Bernard T, Lombard V, Henrissat B: **The Carbohydrate-Active EnZymes database (CAZy): an expert resource for glycogenomics**. *Nucleic acids research* 2009, **37**(suppl 1):D233-D238.

3. Waterhouse RM, Zdobnov EM, Tegenfeldt F, Li J, Kriventseva EV: **OrthoDB: the hierarchical catalog of eukaryotic orthologs in 2011**. *Nucleic acids research* 2011, **39**(suppl 1):D283-D288.

4. Wishart DS, Knox C, Guo AC, Cheng D, Shrivastava S, Tzur D, Gautam B, Hassanali M: **DrugBank: a knowledgebase for drugs, drug actions and drug targets**. *Nucleic Acids Res* 2008, **36**(Database issue):D901-906.

5. Bairoch A: **The ENZYME database in 2000**. *Nucleic Acids Res* 2000, **28**(1):304-305.

6. Finn RD, Mistry J, Tate J, Coggill P, Heger A, Pollington JE, Gavin OL, Gunasekaran P, Ceric G, Forslund K *et al*: **The Pfam protein families database**. *Nucleic Acids Res* 2010, **38**(Database issue):D211-222.

7. Muller J, Szklarczyk D, Julien P, Letunic I, Roth A, Kuhn M, Powell S, Von Mering C, Doerks T, Jensen L: **eggNOG v2. 0: extending the evolutionary genealogy of genes with enhanced non-supervised orthologous groups, species and functional annotations**. *Nucleic acids research* 2010, **38**(suppl 1):D190-D195.

8. Klimke W, Agarwala R, Badretdin A, Chetvernin S, Ciufo S, Fedorov B, Kiryutin B, O’Neill K, Resch W, Resenchuk S: **The national center for biotechnology information's protein clusters database**. *Nucleic acids research* 2009, **37**(suppl 1):D216-D223.

9. Croft D, O’Kelly G, Wu G, Haw R, Gillespie M, Matthews L, Caudy M, Garapati P, Gopinath G, Jassal B: **Reactome: a database of reactions, pathways and biological processes**. *Nucleic acids research* 2011, **39**(suppl 1):D691-D697.

10. Duret L, Mouchiroud D, Gouy M: **HOVERGEN: a database of homologous vertebrate genes**. *Nucleic acids research* 1994, **22**(12):2360-2365.

11. Saier MH, Tran CV, Barabote RD: **TCDB: the Transporter Classification Database for membrane transport protein analyses and information**. *Nucleic acids research* 2006, **34**(suppl 1):D181-D186.

12. Lew JM, Kapopoulou A, Jones LM, Cole ST: **TubercuList–10 years after**. *Tuberculosis* 2011, **91**(1):1-7.

13. Hunter S, Apweiler R, Attwood TK, Bairoch A, Bateman A, Binns D, Bork P, Das U, Daugherty L, Duquenne L *et al*: **InterPro: the integrative protein signature database**. *Nucleic Acids Res* 2009, **37**(Database issue):D211-215.

14. Kanehisa M, Goto S: **KEGG: kyoto encyclopedia of genes and genomes**. *Nucleic Acids Res* 2000, **28**(1):27-30.

15. Chatr-Aryamontri A, Ceol A, Palazzi LM, Nardelli G, Schneider MV, Castagnoli L, Cesareni G: **MINT: the Molecular INTeraction database**. *Nucleic acids research* 2007, **35**(suppl 1):D572-D574.
